# Enzootic stability of tick fever in Holstein calves grazing in a tropical region, subjected to strategic cattle tick control with fluralaner

**DOI:** 10.1186/s13071-024-06212-w

**Published:** 2024-03-10

**Authors:** Dina Maria Beltran Zapa, Lidia Mendes de Aquino, Luiz Felipe Monteiro Couto, Luciana Maffini Heller, Igor Maciel Lopes de Morais, Vanessa Ferreira Salvador, Luccas Lourenzzo Lima Lins Leal, Artur Siqueira Nunes Trindade, Warley Vieira de Freitas Paula, Nicolas Jalowitzki de Lima, Lorena Lopes Ferreira, Daniel de Castro Rodrigues, Tom Strydom, Siddhartha Torres, Vando Edésio Soares, Caio Marcio de Oliveira Monteiro, Felipe da Silva Krawczak, Welber Daniel Zanetti Lopes

**Affiliations:** 1 Center of Veterinary Parasitology, School of Veterinary Science and Animal Science, Federal University of Goiás, Goiânia, Goiás Brazil; 2 Department of Preventive Veterinary Medicine, School of Veterinary and Animal Science, Federal University of Goiás, Goiânia, Goiás Brazil; 3 Department of Preventive Veterinary Medicine, School of Veterinary Medicine, Federal University of Minas Gerais, Belo Horizonte, Minas Gerais Brazil; 4 MSD Animal Health, Franca, São Paulo Brazil; 5 MSD Animal Health, 20 Spartan Road, Isando, Kempton Park, 1619 South Africa; 6 Merck Animal Health, 2 Giralda Farms, Madison, NJ 07940 USA; 7 University of Brazil, Descalvado, São Paulo, Brazil; 8 Department of Biosciences and Technology, Institute of Tropical Pathology and Public Health, Federal University of Goiás, Goiânia, Goiás Brazil

**Keywords:** Anaplasmosis, Babesiosis, Isoxazoline, *Rhipicephalus microplus*

## Abstract

**Background:**

In 2022, fluralaner was launched on the market for use in the control of the cattle tick *Rhipicephalus microplus* after showing 100% efficacy in registration trials against the causative agents of cattle tick fever (TFAs). The aim of the present study was to determine whether a strategic control regimen against *R. microplus* using fluralaner (FLU) in Holstein calves grazing in a tropical region would alter the enzootic stability status of cattle tick fever, triggering outbreaks in these animals up to 22 months age.

**Methods:**

In this study, a group of calves treated with FLU was compared with a control group treated with the regimen currently being used on the farm, which consisted of the fipronil + fluazuron formulation (FIFLUA). In the first experiment, the efficacy of the FIFLUA pour-on formulation was evaluated in a field study. In the second experiment, which lasted 550 days, two experimental groups (*n* = 30/group) of Holstein calves naturally infested with *R. microplus* were analyzed. Calves aged 4 to 10 months received either a specific treatment regimen with FLU (experimental group) or FIFLUA (control group). During this period, tick counts, animal weight measurement, feces collection (to determine eggs and oocysts per gram of feces), tick fever monitoring, blood smears (to ascertain enzootic stability of the herd), PCR testing for TFAs and serology (indirect enzyme-linked immunosorbent assay [iELISA]) were performed. All calves were evaluated for signs of tick fever between ages 11 and 22 months.

**Results:**

FIFLUA showed an acaricidal efficacy of > 90% from post-treatment days 14 to 35. Regarding treatments against the TFAs, the average number of treatments was similar between groups, but animals treated with FLU had a smaller reduction in packed cell volume on some of the evaluation dates of the second and third treatment against TFAs. In calves aged 10 months in the FLU group, *B. bovis* was not detected by PCR (0/15 samples), 40% of the samples had antibody titers and 33% (10/30) of the samples had positive blood smears. Regarding *B. bigemina*, > 86% of the samples in both groups tested positive for *B. bigemina* DNA and antibodies; there was no difference in the antibody titers between the groups. There were no clinical cases of cattle tick fever in calves aged 11 to 22 months.

**Conclusions:**

In comparison with the control treatment, the strategic control regimen against *R. microplus* with FLU that was implemented in the present study did not negatively affect the enzootic stability status of *A. marginale* and *B. bigemina* in the herd up to 22 months of age. The enzootic stability status of *B. bovis* was not reached by either group. These results likely represent a characteristic of the local tick population, so further studies should be performed.

**Graphical Abstract:**

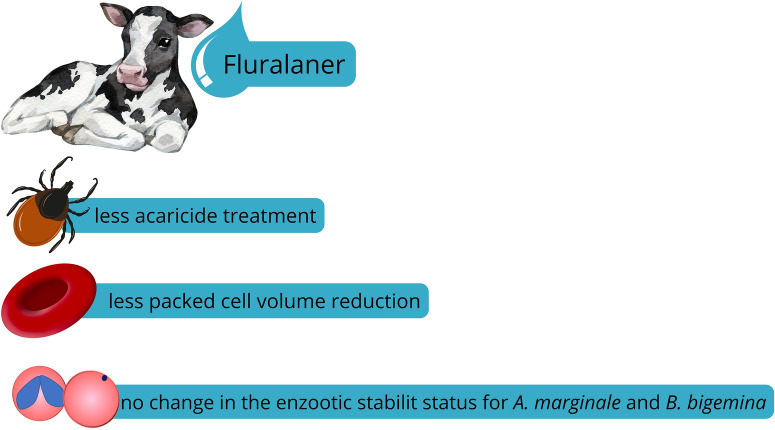

## Background

The cattle tick *Rhipicephalus microplus* is the main vector of *Anaplasma marginale*, *Babesia bovis* and *Babesia bigemina*, which are the causative agents of a syndrome commonly known as tick fever (TF). The causative agents of cattle tick fever (TFAs) and *R. microplus* are closely intertwined in tropical and subtropical regions where this ectoparasite occurs. These tick-borne pathogens cause significant production losses, and depending on animal age, these three causative agents are among the greatest challenges (if not the greatest) in productive cattle breeding, causing considerable morbidity and mortality [1].

To mitigate TF in areas where the disease is endemic, veterinarians and producers implement control strategies against *R. microplus* ticks [2–4]. Although the search for alternative methods to control this tick species is constantly evolving [3–7], the use of synthetic chemicals currently remains the most effective strategy [8–11]. However, constant and intensive use of synthetic acaricides can decrease the efficacy of these chemicals due to resistance selection [12–18]. This has led to the pharmaceutical industry constantly investigating new acaricidal molecules that are efficacious and economically feasible. In 2022, after approximately 30 years without any new products against *R. microplus*, fluralaner (Exzolt®; MSD Animal Health, Merck & Co., Rahway, NJ, USA), belonging to the isoxazoline class, was launched onto the market after achieving 100% efficacy in the initial trials [19]. The introduction of fluralaner (FLU) as a commercial product opened new possibilities for the strategic control of *R. microplus*, but also raised the question of whether one product with such high efficacy could affect the enzootic stability (herd immunity) of cattle in terms of TF.

Enzootic stability usually occurs due to cattle coming into contact with TFA-infected *R. microplus*, resulting in a certain TFA transmission rate that is sufficient to immunize most calves. As a consequence, the immunized calves will not present clinical signs of TF when they become adults. To this end, according to Mahoney and Ross [20], an infection rate > 75% at or before 9 months of age indicates enzootic stability in adult cattle for both *A. marginale* and *Babesia* spp. According to Smith et al. [21], despite the recognized role of ticks in establishing and maintaining herd immunity to TFAs, few studies have evaluated the effect of tick burden and control strategies on the enzootic stability of TFAs in dairy calves subjected to a specific cattle tick control strategy with FLU. Therefore, in the present study, we compared calves treated with FLU with those treated with the fipronil + fluazuron formulation (FIFLUA).

## Methods

### Experimental location and design

The experiments were conducted on a commercial farm (Céu Azul) located in the municipality of Silvânia, Goiás State, Brazil, from January 2022 to July 2023. The region where the farm is located is composed predominantly of Cerrado biome and has a tropical climate. There are two well-defined yearly seasons: rainy summer (October– April), with a mean annual precipitation of 1541 mm, and dry winter (May–September), with rainfall of 150–200 mm, consistent with the “Aw” classification of Köppen-Geiger [22]. The land of the farm comprises plateaus, providing a relatively flat territory, and Holstein cattle (Girolando–Holstein × Gyr, genetic ratio of 31/32 Holstein) are raised on this farm. Up to 24 months of age, the animals are allowed to graze in a pasture where they come into contact with *R. microplus* and TFAs. Soon after calving and during lactation, the cows are placed in a free stall system. During the period when the cows are not producing milk (dry cows), they are released into the pasture, where they again come into contact with *R. microplus* and TFAs.

During the 7 years preceding the study, *R. microplus* control on this farm consisted of the application of a pour-on acaricidal formulation (fipronil + fluazuron [FIFLUA]). There have been no indications of clinical cases of TF in animals after 11 months of age, suggesting the local enzootic stability of TFAs. Since the main objective of this study was to evaluate whether the adoption of a strategic control with FLU against *R. microplus* could alter the enzootic stability, it was necessary to initially verify the susceptibility of the tick strain to the acaricide already used and define a control group. Therefore, a field study was performed (Experiment 1). After confirming that the tick population was susceptible to the product already used by the farm, we performed Experiment 2.

**Fig. 1:**
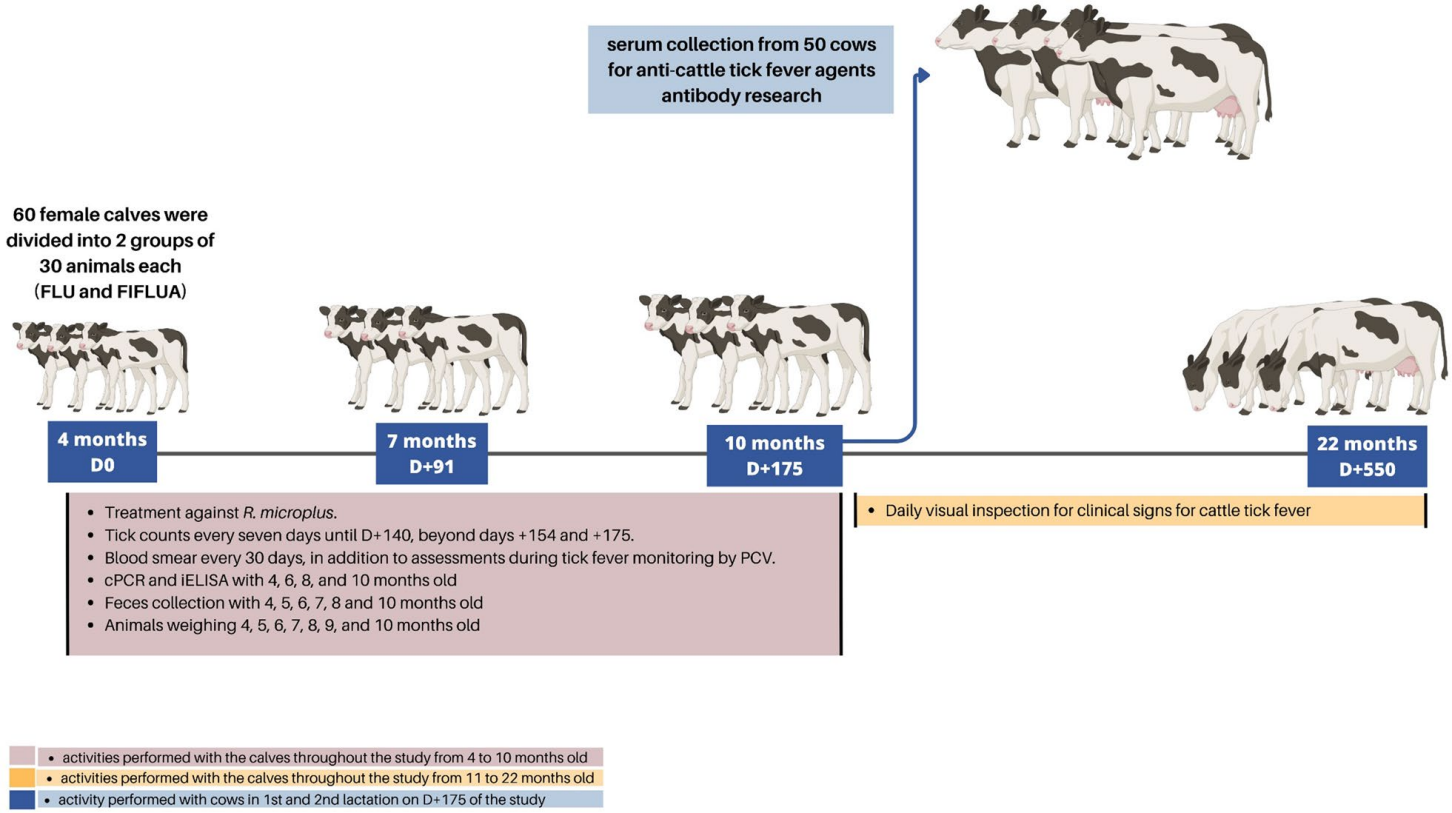
Experimental design of Experiment 2 to evaluate the enzootic stability of cattle tick fever in the herd after treatment with FLU in animals naturally infested with *Rhipicephalus microplus*. D, Study day; FIFLUA, fipronil + fluazuron group; FLU, fluralaner group; iELISA, indirect enzyme-linked immunosorbent assay; PCV, packed cell volume

In Experiment 2, which lasted 550 days, one group of animals was treated with FLU and another group (control) was treated with the pour-on product (FIFLUA) currently being used on the farm. The strategic control regimen against *R. microplus* was implemented in calves aged 4 to 10 months. During this period, tick counts, animal weight measurements and fecal collection (to determine eggs and oocysts per gram of feces) were performed. In addition, we monitored the occurrence of TF and determined the enzootic stability of the herd using PCR and serological tests for TFAs. Blood smears were also examined, but the results were not considered for the classification of enzootic stability because of the lower sensitivity of this technique compared to PCR and serology. According to Mahoney and Ross [20], a *Babesia* spp. exposure rate (serology) > 75% at or before 9 months of age indicates enzootic stability of babesiosis in adult cattle. Although there has been no specific study on *A. marginale*, we extrapolated the concept to this pathogen. The animals in both groups were visually inspected between 11 to 22 months of age for any clinical signs of TF that occurred after the implementation of the strategic control regimen. Serum samples were collected from cows between the first and second lactation to evaluate enzootic stability in this animal stage. Figure 1 summarizes the design of Experiment 2.

### Experiment 1: *R. microplus* strain susceptibility to FIFLUA

Twenty clinically healthy female calves aged 4 to 8 months that were naturally infested with *R. microplus* were divided into two groups of 10 animals each. One group consisted of untreated calves (control group) and one group were treated with the FIFLUA pour-on formulation (fipronil 1.25 mg/kg + fluazuron 2.5 mg/kg; Tick Gard®, MSD Animal Health, Merck & Co.). The methodology procedures used in Experiment 1 were the same as those adopted by Maciel et al. [15].

### Experiment 2: Evaluation of enzootic stability using FLU to control *R. microplus*

Sixty female Holstein calves with a mean age of 4 months were selected from a herd of approximately 400 animals. At the beginning of the study, these calves were allowed to graze in a pasture naturally infested with *R. microplus* and exposed to TFAs, but they had never received any acaricidal treatment. Before the beginning of the study, until weaning at up to 90–100 days of age, the animals were raised in a tropical system and were in contact with soil, grass (Tifton), *R. microplus* and TFAs. The calves received 6 l of cow's milk and 4 kg of feed daily, and had ad libitum access to grass and water. After weaning and up to the beginning of the study, all animals were allocated to the same paddock where they were exposed to *R. microplus* and TFAs.

On day 0 of the experiment, when the animals were 4 months old, the 60 calves were divided into two groups of 30 animals each based on the body weight of each animal, with one group receiving a specific treatment regimen with pour-on FLU (2.5 mg/kg) (Exzolt® 5%; MSD Animal Health, Merck & Co.) (FLU group; experimental group) and the second group receiving a pour-on formulation containing fipronil (1.25 mg/kg) + fluazuron (2.5 mg/kg) (Tick Gard®; MSD Animal Health (FIFLUA group; control group) . The age of each animal, packed cell volume (PCV), body weight and *R. microplus* count (females ≥ 4.5 mm in length) present on the left side of each animal were recorded, according to a method adapted (without multiplying by 2) from Wharton and Utech [23]. The number of animals with ticks < 4 mm between the legs or dewlap was also considered in the formation of the groups. After randomization, the groups were homogeneous in terms of mean age in days (FLU group = 129.1 ± 13.12; FIFLUA group = 130.3 ± 12.11), PCV (FLU group = 27.0% ± 5.2%; FIFLUA group = 27.63% ± 5.6%), live body weight (FLU group = 137.2 ± 18.65 kg; FIFLUA group = 137.2 ± 17.9 kg) and tick count (FLU group = 0.1 ± 0.24; FIFLUA group = 0.2 ± 0.5).

After day 0, the area used was divided into two paddocks of practically identical size and availability of grass and other plant cover. Each group was kept separate from the other throughout the experimental period. The animals in each group received approximately 1% of their live weight of feed per day, in addition to corn silage and water ad libitum. At the beginning of the study, the stocking rate of each experimental area was 8.7 animal units per hectare (au/ha). At the end of the study, the stocking rates for the FLU and FIFLUA groups were 19.0 and 18.5 au/ha, respectively.

### Strategic control schemes against *R. microplus* adopted for the FLU and FIFLUA groups

For the FLU group, FLU was applied on day 0 of the study, when the infestation by *R. microplus* was low (mean 0.1/ tick/animal). The animals were retreated with the FLU formulation only when ticks < 4 mm were observed on ≥ 30% (9/30) of the animals in this group, following the method described by Nicaretta et al. [3, 4]. The animals in the FIFLUA group were retreated with the formulation containing fluralaner only when ticks < 4 mm were observed on ≥ 30% (5/15) of the animals.

On day 0 of the study (D0), infestation by *R. microplus* was low (mean 0.1/tick/animal), and < than 30% of the herd had ticks (≤ 4 mm in length) between the legs and/or in the dewlap region. The first treatment occurred on day 14 of the study (D+14). The same visual inspection criterion was adopted for the animals of this group throughout the experiment until they reached a mean age of 10 months. Regardless of infestation rate, all 30 calves were treated whenever an acaricidal treatment was scheduled.

For the FIFLUA group, the fipronil + fluazuron product was applied according to the manufacturer’s instructions. When ticks were present between the legs and in the dewlap region, farmhands treated the animals when they thought it was necessary. As in the FLU group, when treatment occurred, all animals in the group were treated.

On each day of treatment, cattle in both groups (FLU and FIFLUA) were individually weighed to calculate the correct dosage. The scales used for weighing the animals had been previously tested using a known weight and verified for accuracy. To ensure that dosing techniques were conducted using similar standards, the measured volumes in each experiment were calculated using the individual weight of each animal for each treatment; if necessary, the weight was rounded down to the nearest 0.1 ml. For example, an animal of 177 kg, which would receive 17.7 ml of a pour-on product, received 17.6 ml. Cattle treated with pour-on formulations were not exposed to rain in the first 72 h after each treatment.

### Tick counts, animal weights and feces collection

For both groups, *R. microplus* females (length: 4.5–8 mm) present on the left side of each animal were counted on study days 7, 14, 21 and 28 (D+7, D+14, D+21, D+28, respectively) and then weekly until study day 140 (D+140), in addition to study days 154 and 175 (D+154 and D+175, respectively), according to the method adapted (without multiplying by 2) from Wharton and Utech [23]. At these same time points, the number of animals per group with ticks ≤ 4 mm in length between the legs was quantified [3, 10], registered as present or absent.

The animals were weighed individually on D+0, D+35, D+70, D+98, D+126, D+154 and D+175. Weight gain was calculated for each animal as the difference in body weight during the study, with the animals weighed on scales that had been tested and assessed for accuracy. On D+0, D+35, D+70, D+102, D+130 and D+175, approximately 100–150 g of feces was collected directly from the rectum of each animal. Eggs per gram of feces (EPG) and oocysts of *Eimeria* spp. per gram of feces (OPG) were determined using the technique described by Gordon and Whitlock as modified by Ueno and Gonçalves [24, 25], using a McMaster slide. For animal welfare reasons, although gastrointestinal helminths and *Eimeria* spp. were not the focus of this study, when the degree of infection by any of these agents was ≥ 300 [11], all calves in both groups received specific treatments against these agents with a formulation containing fenbendazole 5 mg/kg + toltrazuril 15 mg/kg (Panacoxx®; MSD Animal Health, Merck & Co.).

### Cattle TF monitoring and rescue treatment against TFAs

Packed cell volume monitoring and rescue treatment for TFAs followed the method described by Heller et al. [2]. For both groups (FLU and FIFLUA) in Experiment 2, PCV was measured using the microhematocrit technique of Weiss and Wardrop [26] on D+0, D+4, D+7 and D+11 and then every 3 days until D+140, in addition to D+154 and D+175. Approximately 4 ml of blood was collected from the coccygeal vein of each animal into tubes containing EDTA (K2 EDTA; BD Vacutainer®; BD, Franklin Lakes, NJ, USA), from which capillary tubes were filled, followed by centrifugation (13,000 *g* for 5 min) to evaluate PCV using an appropriate scale [27].

If the PCV value for a calf decreased by > 4 percentage points compared to the last assessment date (for example, 32% to 27%, considering the assessment of 2 samples) or decreased by > 5 percentage points compared to the two last evaluation dates (e.g., 32% to 29% and then to 26% considering the evaluation of 3 samples), the animal was treated subcutaneously with 3.5 mg/kg diminazene (Ganazeg®; Elanco Animal Health, Indianapolis, IN, USA) and 20 mg/kg of oxytetracycline intramuscularly (Oxitrat® Plus; MSD Animal Health, Merck & Co.) [2].

To determine the etiological agent involved, on each date that one calf received treatment against TFAs, cytological examination for *A. marginale*, *B. bigemina* and *B. bovis* were performed. Smears using blood collected from the tip of the tail and stained with Giemsa were examined with an optical microscope (1000× magnification). The percentage of parasitemia (*Babesia* spp.) or bacteremia (*A. marginale*) was calculated following the method described by the Inter-American Institute for Cooperation on Agriculture (IICA) [28] and Coetzee et al. [29].

### Evaluation of the enzootic stability of TF: PCR, indirect enzyme-linked immunosorbent assay and blood smears

On D+0, D+42, D+154 and D+175 of Experiment 2, 15 animals were randomly chosen from each group for PCR testing for TFAs. Blood samples were subjected to DNA extraction using the DNA Mini Spin Kit (KASVI; São José dos Pinhais, PR, Brazil), following the manufacturer’s instructions, and the DNA was tested in three different PCR assays, targeting *A. marginale*, *B. bovis* and *B. bigemina*, respectively.

The PCR assays targeted a 458-bp fragment of the major surface protein 5 (msp5) gene of *A. marginale* [30, 31], a 356-bp fragment of the rhoptry-associated protein 1a (Rap-1a) gene of *B. bovis* [32] and an approximately 440-bp fragment of the variant erythrocyte surface antigen (ves-1α) gene of *B. bigemina* [33]. Negative control (PCR-grade water, Sigma-Aldrich, St. Louis, MO, USA) and an appropriate positive control sample (DNA of *B. bovis*, *B. bigemina* or *A. marginale*) were run together with the cattle DNA samples. Negative samples were further tested using PCR protocols targeting the cytochrome* b* gene (cytB) of mammals [34] to validate the DNA extraction protocol. If a sample did not produce any product in these PCR assays, the sample was discarded from the analysis. PCR products were stained with SYBR Safe (Invitrogen, Thermo Fisher Scientific, Waltham, MA, USA), following the manufacturer’s recommendations, and were visualized by electrophoresis in 1.5% agarose gel with an ultraviolet transilluminator.

Serum samples were also collected (D+0, D+42, D+154 and D+175) from the 15 randomly selected animals and tested for immunoglobulin G (IgG) antibodies against *A. marginale*,* B. bovis* and *B. bigemina* using an indirect enzyme-linked immunosorbent assay (iELISA) following the protocol described by Andrade et al. [35] and Machado et al. [35]. On D+0, D+35, D+70, D+102, D+130 and D+175, cytological examination for *A. marginale*, *B. bigemina* and *B. bovis* was performed via blood smears for all 60 animals (i.e. both groups), as described in the section Cattle TF monitoring and rescue treatment against TFAs. In addition, the stained smears prepared on the day of each rescue treatment were added to these results.

After completing treatment to an average age of 10 months, the evaluations were stopped, and the animals were returned to their normal farm routine. The animals were visually inspected daily (apathy, drooping eyelids and ears) for possible clinical signs of TF up to 22 months of age. If there was any clinical suspicion of TF during this period (11–22 months of age), blood was collected for PCV, blood smear, PCR and iELISA testing.

To evaluate the enzootic stability of TFAs in cows on the farm, serum samples were obtained from 50 cows between their first and second lactation on D+175 and tested for IgG against *A. marginale*,* B. bovis* and *B. bigemina* using iELISA, as previously described. For each procedure, a different needle and syringe were used for each animal.

### Statistical analyses

The data on tick counts, PCV, EPG, OPG and serological tests did not meet the assumptions of normality, homogeneity of variance, residuals and randomness, even after log(count + 1) transformation. Therefore, the experimental groups were compared using the Kruskal–Wallis test. The mean number of treatments against TFAs performed on cattle per group at 3–10 months was also analyzed using the Kruskal‒Wallis test. These treatments were also analyzed per animal in blocks (first, second, third block post initiation of the study, up to the maximum treatment that an animal received) in relation to the order of occurrence for each animal and each group.

Live body weight (LBW) and live body weight gain (LBWG) were submitted to analysis of covariance, with the observations on D+0 for LBW and LBWG from D+0 to D+35 as covariables. Treatment means were compared using the F-test.

All statistical procedures were performed using the software Statistical Analysis System (SAS), version 9.4 [36]. Differences were considered statistically significant when *P* < 0.05.

## Results

In Experiment 1, on D+0 there was no difference (Kruskal–Wallis H-test, *H* = 0.03, *df* = 1, *P* = 0.9542) in mean tick counts for the treated and control groups. However, from the 7th day post-treatment (DPT) until the 49th DPT, the parasite load was lower in the treated group than in the control group. Efficacy ranged from 72.1 to 97.0%, with values > 90% between 14 and 35 DPT (Table 1).

**Table 1:** Mean count of female *Rhipicephalus microplus* (≥ 4.5 mm) in naturally infested cattle treated or not with the fipronil + fluazuron formulation (FIFLUA), as an indicator of treatment efficacy

Day of the study	Control group (untreated)		Treated group (FIFLUA)		*P* value	Coefficient of variation	Efficacy (%)
	Mean count^1^	Range	Mean count^1^	Range			
0^*, x^	56.90 a	36.64–75.27	56.7 a	36.33–76.03	0.9542	21.13	**_**
7	52.64 a	28–88	12.40 b	8–45	< 0.0001	19.85	76.36
14	42.67 a	32–91	2.10 b	0–8	< 0.0001	17.64	95.06
21	72.50 a	16–75	3.20 b	0–7	< 0.0001	24.36	95.57
28	87.60 a	29–98	2.60 b	0–5	< 0.0001	21.57	97.02
35	62.30 a	24–78	5.20 b	0–6	< 0.0001	23.67	91.62
42	72.60 a	36–112	12.40 b	2–12	< 0.0001	24.38	82.86
49	88.40 a	24–124	24.60 b	16.0–58.0	0.0003	24.62	72.07

^1^Mean count values followed by the same letter in the same row do not differ significantly at a 95% reliability level of significance

^*^Mean counts of *Rhipicephalus microplus* females on days - 3, - 2, and - 1

^x^Treatment

**Fig. 2:**
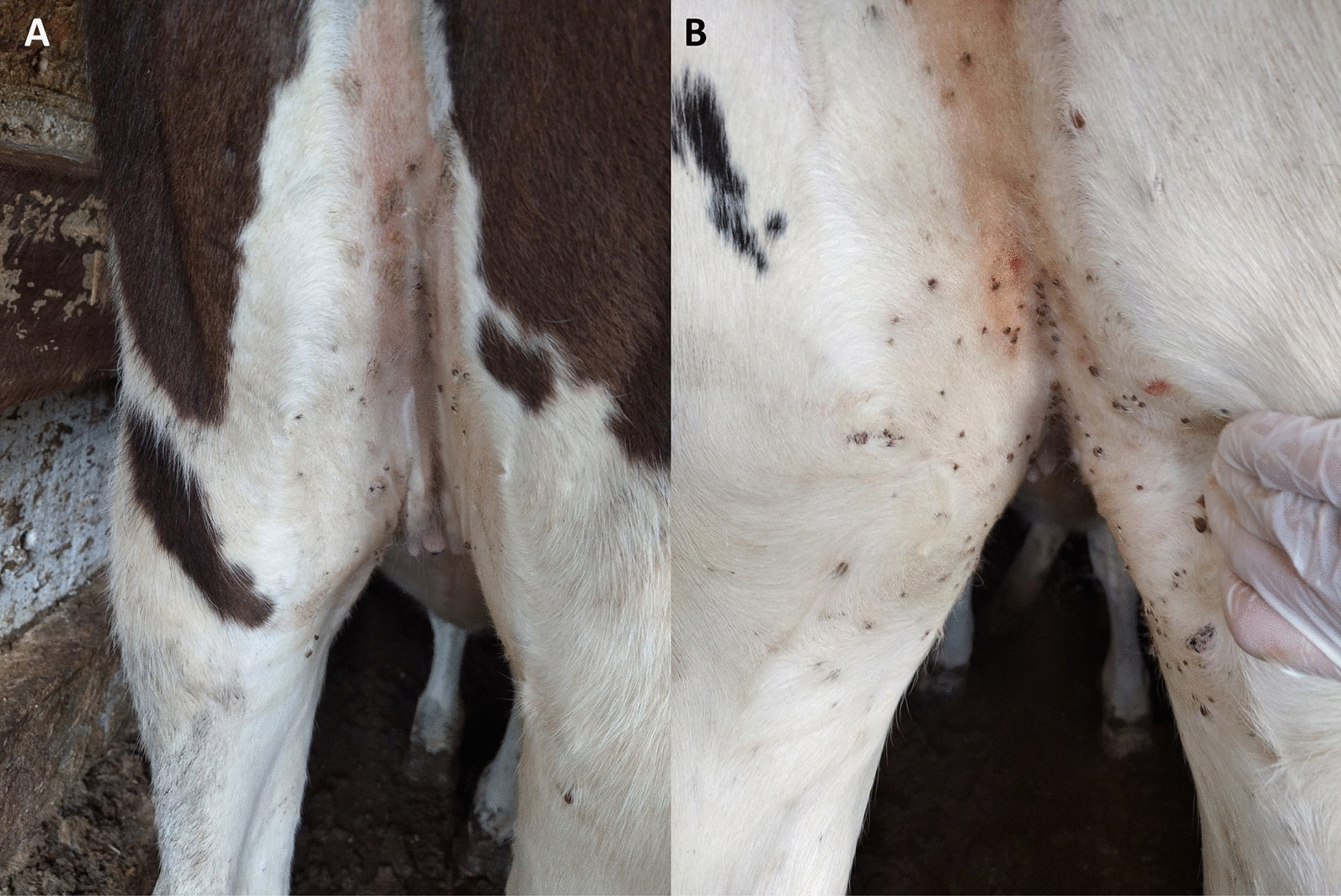
Ticks between the legs of cattle in the group treated with fluralaner. **A** Animal 5411 on study day 63 when treated. **B** animal 5359 on study day 133 when treated again

During Experiment 2 and following the criteria established in this experiment, three acaricidal treatments were performed in the FLU group and four treatments were performed in the FIFLUA animals. After the first treatment of FLU, retreatments with FLU occurred at intervals of 49 and 70 days between applications. In the FIFLUA group, after the first treatment, three retreatments occurred at intervals of 28, 42 and 42 days (Table 2). All animals between 4 and 10 months of age from both groups came into contact with *R. microplus* < 4 mm in length (Table 2; Fig. 2). Of the 23 tick count dates, on 12 days (49, 56, 70, 84, 91, 98, 105, 112, 119, 126, 154 and 175) the mean *R. microplus* counts were lower in the FLU group than in the FIFLUA group (Table 2).

**Table 2:** Number of animals infested with ticks < 4 mm in length between the legs of the animal or in the dewlap region and average counts of female *Rhipicephalus microplus* (≥ 4.5 mm in length) present on the left side of the body of animals subjected to different control schemes against *R. microplus*

		Number of animals with ticks < 4 mm in lenght (%)		Tick counts (females (≥4.5 mm in lenght)										Value of *P*
Day	Animal age in months			Treatment (FLU)					Control (FIFLUA)					
		Treatment (FLU)	Control (FIFLUA)	Mean*		Range			Mean*		Range			
0	4	6/30 (20)	6/30 (20)	0.03	^A^	0	–	1	0.23	^A^	0	–	2	0.0761
7		7/30 (23.3)	6/30 (20)	0.19	^A^	0	–	2	0.20	^A^	0	–	4	0.7248
14^αβ^		13/30 (43.3)	14/30 (46.6)	0.00	^A^	0	–	0	0.03	^A^	0	–	1	0.3094
21		0/30 (0)	1/30 (3.3)	0.00	^A^	0	–	0	0.03	^A^	0	–	1	0.3094
28		5/30 (16.6)	6/30 (20)	0.00	^A^	0	–	0	0.00	^A^	0	–	0	1.0000

35	5	0/30 (0)	2/30 (6.6)	0.00	^A^	0	–	0	0.10	^A^	0	–	1	0.0733
42 ^β^		0/30 (0)	28/30 (93.3)	0.00	^A^	0	–	0	0.07	^A^	0	–	2	0.3094
49		2/30 (6.6)	21/30 (70)	0.00	^B^	0	–	0	0.83	^A^	0	–	7	0.0011
56		7/30 (23.3)	20/30 (66.6)	0.00	^B^	0	–	0	0.87	^A^	0	–	5	< 0.0001

63 ^α^	6	20/30 (66.6)	21/30 (70)	0.65	^A^	0	–	8	0.73	^A^	0	–	5	0.3237
70		0/30 (0)	18/30 (60)	0.03	^B^	0	–	1	1.00	^A^	0	–	6	0.0009
77		1/30 (3.3)	12/30 (40)	0.00	^A^	0	–	0	0.00	^A^	0	–	0	1.0000
84 ^β^		0/30 (0)	26/30 (86.6)	0.00	^B^	0	–	0	0.20	^A^	0	–	2	0.0187

91	7	0/30 (0)	19/30 (63.3)	0.00	^B^	0	–	0	0.97	^A^	0	–	8	0.0023
98		0/30 (0)	24/30 (80)	0.00	^B^	0	–	0	0.67	^A^	0	–	5	0.0002
105		0/30 (0)	30/30 (100)	0.00	^B^	0	–	0	1.93	^A^	0	–	8	< 0.0001
112		0/30 (0)	22/30 (73.3)	0.00	^B^	0	–	0	0.43	^A^	0	–	6	0.0188

119	8	0/30 (0)	23/30 (76.6)	0.03	^B^	0	–	1	0.60	^A^	0	–	3	0.0010
126 ^β^		8/30 (26.6)	29/30 (96.6)	0.03	^B^	0	–	1	0.70	^A^	0	–	5	0.0010
133 ^α^		29/30 (96.6)	22/30 (73.3)	0.39	^A^	0	–	4	1.00	^A^	0	–	9	0.4115
140		0/30 (0)	30/30 (100)	1.29	^A^	0	–	5	0.90	^A^	0	–	4	0.3342

154	9	0/30 (0)	24/30 (80)	0.00	^B^	0	–	0	0.70	^A^	0	–	6	< 0.0001

175	10	5/30 (16.6)	27/30 (90)	0.00	^B^	0	–	0	0.90	^A^	0	–	11	< 0.0001

*Mean count values followed by the same letter in the same row do not differ significantly at a 95% reliability level of significance (Kruskal–Wallis test)

*FIFLUA* Fipronil + fluazuron formulation,* FLU* fluralaner formulation

^α^All 30 animals in FLU group received fluralaner

^β^All 30 animals in FIFLUA group received fipronil + fluazuron

An average of 5.1 and 5.0 rescue treatments per animal (Kruskal–Wallis H-test, *H* = 0.14, *df* = 1, *P* = 0.7057) were performed against TFAs in the FLU and FIFLU groups, respectively. On D+7 after the second rescue treatment (Kruskal–Wallis H-test, *H* = 4.94, *df* = 1, *P* = 0.0272) and on D+3 (Kruskal–Wallis H-test, *H* = 11.61, *df* = 1, *P* = 0.0143) and D+7 (Kruskal–Wallis H-test, *H* = 9.77, *df* = 1, *P* = 0.0150) of the fourth rescue treatment, mean values of *A. marginale* bacteremia were lower in the FLU group than in the FIFLU group. PCV values were, on average, higher in the FLU group than in the FIFLU group on D+7 of the second rescue treatment (Kruskal–Wallis H-test, *H* = 3.74, *df* = 1, *P* = 0.0221) and on D+0 of the third rescue treatment (Kruskal–Wallis H-test, *H* = 6.50, *df* = 1, *P* = 0.0107) (Table 3). There was no difference in parasitemia values between groups for *B. bovis* and *B. bigemina* throughout Experiment 2 (Table 4).

**Table 3:** Summary of the number of treatments against *Anaplasma marginale*, percentage of bacteremia and packed cell volume for calves subjected to the different control schemes against *Rhipicephalus microplus*

Treatment	Variable	Day	FLU	Control (FIFLUA)	*P* value
1st	*A. marginale* bacteremia (%)	0	1.17 (0.0–3.2) a	1.27 (0.2–3.0) a	0.7553
		3	0.55 (0.0–1.4) a	0.63(0.0–2.5) a	0.9398
		7	0.36 (0.0–2.1) a	0.39 (0.0–1.1) a	0.2794
	PCV (%)	0	28.16 (15–38) a	26.67 (13–35) a	0.7047
		3	30.67 (23–40) a	29.59 (11–37) a	0.9335
		7	30.57 (24–41) a	29.97 (18–39) a	0.8468

2nd	*A. marginale* bacteremia (%)	0	1.51 (0.2–3.4) a	1.88 (0.0–4.0) a	0.2215
		3	0.75 (0.0–2.2) a	1.03 (0.0–3.2) a	0.2196
		7	0.32 (0.0–2.0) b	0.61 (0.0–2.1) a	0.0272
	PCV (%)	0	27.1 (15–39) a	23.34 (12–40) a	0.0355
		3	28.80 (19–39) a	25.21 (15–38) a	0.0626
		7	28.97 (11–36) a	26.21 (18–37) b	0.0221

3rd	*A. marginale* bacteremia (%)	0	1.29 (0.2–3.1) a	1.43 (0.0–3.6) a	0.4729
		3	0.61 (0.0–2.1) a	0.76 (0.0–2.2) a	0.4143
		7	0.29 (0.0–1.0) a	0.39 (0.0 -1.3) a	0.1558
	PCV (%)	0	24.14 (15–32) a	21.70 (12–35) b	0.0107
		3	25.61 (16–34) a	23.37 (15–34) a	0.0567
		7	26.52 (18–33) a	25.15 (18–33) a	0.1975

4th	*A. marginale* bacteremia (%)	0	0.87 (0.0–3.4) a	1.44 (0.2–3.5) a	0.1558
		3	0.24 (0.0–2.1) b	0.84 (0.0–2.2) a	0.0143
		7	0.10 (0.0–1.2) b	0.41 (0.0–1.3) a	0.0150
	PCV (%)	0	21.62 (14–28) a	20.67 (10–28) a	0.6746
		3	23.77 (14–30) a	22.05 (9–28) a	0.5761
		7	25.46 (19–31)a	24.95 (14–30)a	0.6971

5th	*A. marginale* bacteremia (%)	0	1.56 (0.4–2.9)a	1.12 (0.0–2.8)a	0.3558
		3	0.46 (0.0–2.3)a	0.39 (0.0–1.2)a	0.7292
		7	0.19 (0.0–0.8)a	0.23 (0.0–0.9)a	0.9571
	PCV (%)	0	21.00 (15–25)a	21.57 (16–26)a	0.2012
		3	23.28 (15–31)a	23.79 (18–29)a	0.3261
		7	26.39 (20–32)a	25.71 (15–33)a	0.9209
Mean number of treatments performed in cattle between 3 to 10 months of age			5.06 (2–8)a	5.03 (2–8)a	0.8596

*FIFLUA* Fipronil + fluazuron formulation,* FLU* fluralaner formulation, *PCV* packed cell volume

Mean values (range given in parentheses) followed by the same letter in the same row do not differ significantly at a 95% reliability level

**Table 4:** Mean parasitemia of *Babesia bigemina* and *Babesia bovis* on three experimental days (0, 3 and 7) after the first 5 treatments against these tick fever agents and the mean number of treatments against these parasites for calves subjected to different control schemes against *Rhipicephalus microplus*

Treatment	Agent	Day	Mean parasitemia, in % (range)		*P *value
			FLU-treated group	Control group (FIFLUA-treated)	
1st	*Babesia bigemina*	0	0.03 (0.0–0.8) a	0.03 (0.0–0.8) a	0.9621
		3	0.00 (0.0–0.0) a	0.00 (0.0–0.0) a	1.000
		7	0.00 (0.0–0.0) a	0.00 (0.0–0.0) a	1.000
	*Babesia bovis*	0	0.00 (0.0–0.0) a	0.00 (0.0–0.0) a	1.000
		3	0.00 (0.0–0.0) a	0.00 (0.0–0.0) a	1.000
		7	0.00 (0.0–0.0) a	0.00 (0.0–0.0) a	1.000

2nd	*Babesia bigemina*	0	0.02 (0.0–0.5) a	0.00 (0.0–0.0) a	0.334
		3	0.00 (0.0–0.0) a	0.00 (0.0–0.0) a	1.000
		7	0.00 (0.0–0.0) a	0.00 (0.0–0.0) a	1.000
	*Babesia bovis*	0	0.02 (0.0–0.6) a	0.00 (0.0–0.0) a	0.1678
		3	0.00 (0.0–0.0) a	0.00 (0.0–0.0) a	1.000
		7	0.00 (0.0–0.0) a	0.00 (0.0–0.0) a	1.000

3rd	*Babesia bigemina*	0	0.03 (0.0–0.5) a	0.00 (0.0–0.0) a	0.5705
		3	0.00 (0.0–0.0) a	0.00 (0.0–0.0) a	1.000
		7	0.00 (0.0–0.0) a	0.00 (0.0–0.0) a	1.000
	*Babesia bovis*	0	0.00 (0.0–0.1) a	0.04 (0.0–1.1) a	0.8666
		3	0.00 (0.0–0.0) a	0.00 (0.0–0.0) a	1.000
		7	0.00 (0.0–0.0) a	0.00 (0.0–0.0) a	1.000

4th	*Babesia bigemina*	0	0.02 (0.0–0.4) a	0.05 (0.0–0.5) a	0.7258
		3	0.00 (0.0–0.0) a	0.00 (0.0–0.0) a	1.000
		7	0.00 (0.0–0.0) a	0.00 (0.0–0.0) a	1.000
	*Babesia bovis*	0	0.04 (0.0–0.7) a	0.03 (0.0–0.6) a	0.5091
		3	0.00 (0.0–0.0) a	0.00 (0.0–0.0) a	1.000
		7	0.00 (0.0–0.0) a	0.00 (0.0–0.0) a	1.000

5th	*Babesia bigemina*	0	0.16 (0.0–0.6) a	0.11 (0.0–0.7) a	0.9672
		3	0.00 (0.0–0.0) a	0.00 (0.0–0.0) a	1.000
		7	0.00 (0.0–0.0) a	0.00 (0.0–0.0) a	1.000
	*Babesia bovis*	0	0.21 (0.0–1.5) a	0.02 (0.0–0.2) a	0.1052
		3	0.00 (0.0–0.0) a	0.00 (0.0–0.0) a	1.000
		7	0.00 (0.0–0.0) a	0.00 (0.0–0.0) a	1.000
Mean number of treatments performed in cattle between 3 to 10 months of age		5.06 (2–8) a		5.03 (2–8) a	0.8596

Mean values (range given in parentheses) followed by the same letter in the same row do not differ significantly at a 95% reliability level

*FIFLUA* Fipronil + fluazuron formulation,* FLU* fluralaner formulation

**Fig. 3:**
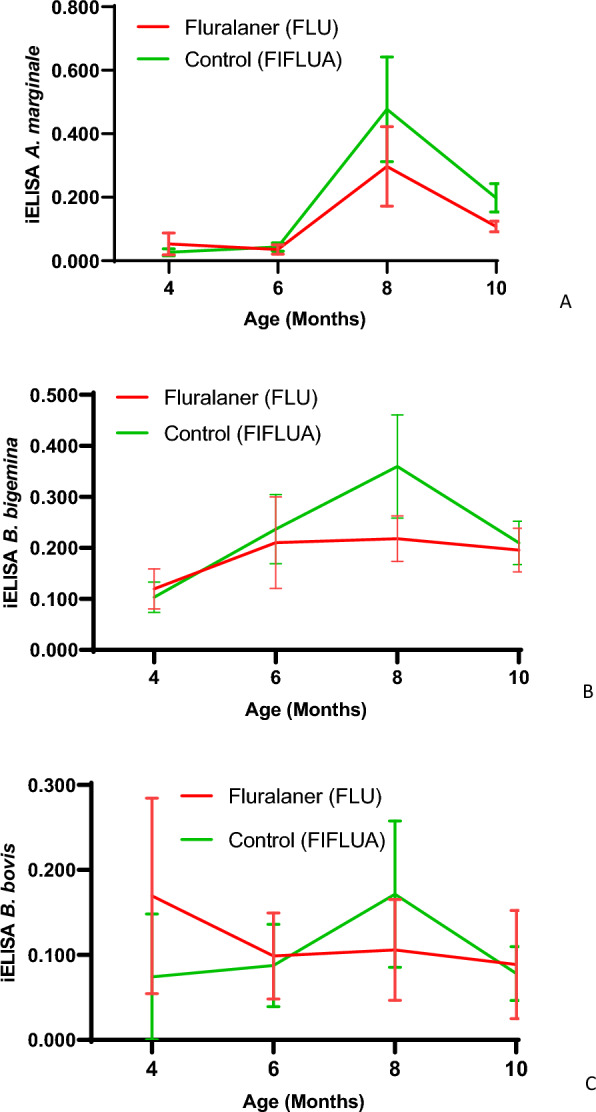
iELISA antibody titers against tick fever agents in cattle treated with fluralaner and a control treatment against the tick *Rhipicephalus microplus*. **a** *Anaplasma marginale*, **b** *Babesia bigemina*, **c** *Babesia bovis*. No significant difference between the FLU and FIFLUA groups was observed (*P* ≥ 0.05). FIFLUA, Fipronil + fluazuron group; FLU, fluralaner group; iELISA, indirect enzyme-linked immunosorbent assay

Complete data for PCR, iELISA and blood smear results are given in Table 5. There was no significant difference in antibody titers between the two groups throughout the study for the three TFAs evaluated (*B. bovis*, *B. bigemina* and *A. marginale*) (Fig. 3).

**Table 5:** Detection of *Anaplasma marginale, Babesia bigemina and Babesia bovis* by means of conventional PCR (DNA), serology (iELISA) and blood smears using blood samples collected from cattle subjected to different control schemes against *Rhipicephalus microplus*

Tick fever agent	Animal age and treatment							
	4 months		6 months		8 months		10 months	
	Treatment (FLU)	Control (FIFLUA )	Treatment (FLU)	Control (FIFLUA)	Treatment (FLU)	Control (FIFLUA)	Treatment (FLU)	Control (FIFLUA)
*PCR*								
*B. bovis*	0/15 (0)	0/15 (0)	2/15 (13.3)	3/15 (20)	0/15 (0)	0/15 (0)	0/15 (0)	1/15 (6.6)
*B. bigemina*	5/15 (33.3)	11/15 (73.3)	9/15 (60)	12/15 (80)	12/15 (80)	15/15 (100)	13/15 (86.7)	15/15 (100)
*A. marginale*	14/15 (93.3)	13/15 (86.6)	15/15 (100)	15/15 (100)	15/15 (100)	15/15 (100)	15/15 (100)	15/15 (100)
*iELISA*								
*B. bovis*	3/15 (20)	1/15 (6.6)	5/15 (33.3)	5/15 (33.3)	4/15 (26.6)	7/15 (46.6)	6/15 (40)	7/15 (46.6)
*B. bigemina*	11/15 (73.3)	11/15 (73.3)	14/15 (93.3)	15/15 (100)	14/15 (93.3)	13/15 (86.6)	15/15 (100)	15/15 (100)
*A. marginale*	4/15 (26.6)	7/15 (46.6)	7/15 (46.6)	8/15 (53.3)	14/15 (93.3)	14/15 (93.3)	15/15 (100)	15/15 (100)
Blood smear^*^								
*B. bovis*	0/30 (0)	0/30 (0)	2/30 (6.6)	0/30 (0)	10/30 (33.3)	7/30 (23.3)	1/30 (3.3)	0/30 (0)
*B. bigemina*	01/30 (3.3)	0/30 (0)	1/30 (3.3)	1/30 (3.3)	14/30 (46.6)	11/30 (36.6)	3/30 (10)	0/30 (0)
*A. marginale*	19/30 (63.3)	17/30 (56.6)	27/30 (90)	30/30 (100)	30/30 (100)	30/30 (100)	30/30 (100)	30/30 (100)

Values in table are presented as the number of animals who tested positive for tick fever agents among the animals tested, with the prevalence (%) given in parentheses

*PCR* Conventional PCR, *FIFLUA* fipronil + fluazuron formulation,* FLU* fluralaner formulation,* iELISA* indirect enzyme-linked immunosorbent assay

^*^Blood smears were performed on all 30 animals in each group

Body weight (analysis of variance [ANOVA], *F*_(1,58)_ = 3.89, *P* = 0.0307) and weight gain (ANOVA, *F*_(1,58)_ = 4.77, *P* = 0.0330) were higher in the FLU group than in the FIFLUA group at 9 months of age (D+154). In addition, there was a trend toward greater weight gain by the animals in the FLU group at 8 months of age, but this difference was not statistically significant (ANOVA, *F*_(1,58)_ = 3.53, *P* = 0.0655) (D+126; Table 6). Regarding gastrointestinal helminths, the mean EPG counts were higher in the FLU group than in the FIFLUA animals (*P* ≤ 0.05) on D+70 (Kruskal–Wallis H-test, *H* = 10.38, *df* = 1, *P* = 0.0013) and D+130 (Kruskal–Wallis H-test, *H* = 6.91, *df* = 1, *P* = 0.0086). There was no difference between the FLU and FIFLUA groups in mean OPG counts throughout the study (Table 6).

**Table 6:** Analysis of covariance of body weight and live weight gain of cattle subjected to different control schemes against *Rhipicephalus microplus* for 175 days

Study day	Animal age (months)	Variable	Experimental groups		Covariance analysis	
			Treated gruoup (FLU)	Control group (FIFLUA)	Pr > F	Pr > Covariate
0	4	Weight	137.26 ± 18.65	137.28 ± 17.95	Covariate	
35	5		150.85 ± 20.87 a	148.83 ± 30.72 a	0.5147	< 0.0001
70	6		189.42 ± 25.27 a	187.45 ± 39.72 a	0.6692	< 0.0001
98	7		213.05 ± 25.33 a	211.26 ± 43.32 a	0.7037	< 0.0001
126	8		242.02 ± 27.81 a	233.00 ± 48.79 a	0.1103	< 0.0001
154	9		276.13 ± 21.62 a	266.16 ± 53.84 b	0.0307	< 0.0001
175	10		299.85 ± 21.40 a	292.93 ± 59.06 a	0.2258	< 0.0001

0–35	5	Weight gain	13.60 ± 11.68	11.56 ± 11.51	Covariate	
0–74	6		52.16 ± 17.05 a	50.18 ± 17.59 a	0.8032	< 0.0001
0–98	7		75.79 ± 15.66 a	73.98 ± 21.32 a	0.8842	< 0.0001
0–126	8		104.76 ± 18.28 a	95.72 ± 27.56 a	0.0655	< 0.0001
0–154	9		138.80 ± 15.07 a	128.88 ± 29.63 b	0.0330	< 0.0001
0–175	10		162.60 ± 17.06 a	155.66 ± 34.90 a	0.2195	< 0.0001

Values in table are presented as the mean ± standard deviation. Means followed by the same letter in the same row do not differ significantly at a 95% reliability level (F-test)

*FIFLUA* Fipronil + fluazuron formulation,* FLU* fluralaner formulation

From 11 to 22 months of age, there were no clinical cases of TF based on daily visual inspections of the cattle. Of the serum samples collected from 50 cows between first and second lactation (D+175), 98% (49/50) contained antibodies against *A. marginale*, 86% (42/50) contained antibodies against *B. bigemina* and 36% (18/50) contained antibodies against *B. bovis*.

## Discussion

This study provides practical information related to the strategic use of pour-on fluralaner against *R. microplus* and its possible impact on TFAs in Holstein calves, in a farm located in a tropical climate region of Brazil (Table 7).

**Table 7:** Multiple comparison results for strongyles eggs and *Eimeria* spp. oocysts in cattle subjected to different groups

EPG counts–Strongyles eggs						
Study day	Animal age (in months)	Treated group (FLU)		Control group (FIFLUA)		*P* value
		Mean	Range	Mean	Range	
0	4	11.29 a	0–150	17.24 a	0–0	0.2942
35	5	90.32 a	0–450	98.28 a	0–100	0.8664
70	6	453.23 a	0–3500	141.07 b	0–0	0.0013
102	7	66.13 a	0–400	87.93 a	0–0	0.3746
130	8	288.65 a	0–1300	105.17 b	0–500	0.0086
175	10	31.68 a	0–250	24.14 a	0–150	0.8783

OPG counts–*Eimeria* spp. oocysts						
Study day	Animal age (in months)	Treated group (FLU)		Control group (FIFLUA)		*P* value
		Mean	Range	Mean	Range	
0	4	3.23 a	0–100	0.00 a	0–0	0.3252
35	5	3.23 a	0–50	3.33 a	0–100	0.6032
70	6	14.52 a	0–450	0.00 a	0–0	0.3252
102	7	0.00 a	0–0	0.00 a	00–	1.0000
130	8	6.68 a	0–	76.67 a	0–500	0.0537
175	10	28.32 a	0–200	18.33 a	0–150	0.4156

Means followed by the same letter in the same row do not differ significantly at a 95% reliability level (Kruskal–Wallis test)

*EPG* Eggs per gram of feces, *FIFLUA* fipronil + fluazuron formulation,* FLU* fluralaner formulation,* OPG* oocysts per gram of feces

In the present study, the percentage of calves treated with FLU presenting antibodies against *B. bigemina* and *A. marginale* reached > 75% at 6 and 8 months of age, respectively, indicating enzootic stability according to Mahoney and Ross [20]. These latter authors recommended that the enzootic stability should be assessed by means of serological analyses. However, at the time Mahoney and Ross [20] conducted their study, PCR testing was not available. Using PCR results, > 75% of the animals treated with FLU were PCR positive for *B. bigemina* and *A. marginale* at 8 and 4 months of age, respectively. Even though at the beginning of the study the infection rate by *A. marginale* in the FLU group was > 75%, it is possible to state that both strategic control schemes against *R. microplus* in the FLU and FIFLUA animals did not prevent the cattle from continuing to be exposed to *A. marginale* and *B. bigemina*. The number of animals infected by these two agents, by both PCR and serological testing, at 4 months of age, was lower than the total number of positive cattle at 10 months of age. This same rationale can be applied to the blood smear technique regarding *A. marginale*, but not to *B. bigemina*. The low prevalence of *B. bovis* and *B. bigemina* using blood smears has also been reported by other authors [2, 65].

We observed higher infection rates of *A. marginale* than *B. bigemina* and for both the FLU- and FIFLUA-treated groups. This finding is consistent with results reported by other authors during the last 15 years, who have described a higher prevalence of anaplasmosis than babesiosis [1, 2, 19, 1, 2]. However, there is still a need for further studies to better understand these findings. In terms of the biological and epidemiological aspects of *A. marginale*, this rickettsia has shown a greater ability to infect cattle than *Babesia* spp. [40–42]. After one infection with *A. marginale*, animals can become persistently infected for life [43], while this has not been reported for *Babesia* spp. [44]. *Anaplasma marginale* may exhibit genetic diversity, which can increase the frequency of clinical cases [1, 45–47]. Reports of the ineffectiveness of products used for the treatment of TFAs are more frequent for *A. marginale* than for *Babesia* spp. [48–50], explaining why it is more difficult to control infection by this rickettsia using chemical products.

Regarding our results for *B. bovis*, 40% (6/15) and 46.6% (7/15) of the animals treated with FLU and the control regimen, respectively, had antibodies against this parasite at 10 months of age. Based on the definition of enzootic stability described by Mahoney and Ross [20], both groups showed enzootic instability for *B. bovis*. The authors of studies in the same region approximately 22 years ago reported that the prevalence of TFAs was > 75% [51–53]. However, recent studies have reported the incidences of *B. bovis* and *B. bigemina* to be < 75%, characterizing enzootic instability [20]. Using nested-PCR (nPCR), Bahia et al. [39] evaluated dairy calves at the same age as the animals in the present study and found prevalences of 4–6.6% for *B. bovis* and 12–14% for *B. bigemina*. Martins et al. [54] evaluated Nellore crossbred calves aged between 10 and 22 months for a 1-year period, without the use of acaricides. In that study, all samples tested by both conventional and real-time PCR were positive for *B. bigemina*, with no samples positive for *B. bovis*. The authors conducted serology only for *B. bigemina,* and the prevalence was 13% and 15% for Brangus and Nellore cattle, respectively [54].

In the field, it is possible that enzootic stability is more likely for *B. bigemina* than for *B. bovis* in regions where both are present [44], and some theories have been elaborated to explain this hypothesis. The rate of infection in cattle ticks has been found to be higher for *B. bigemina* than for *B. bovis* [55–60] and, consequently, the degree of parasitemia in cattle in the field tends to be higher for *B. bigemina* than for *B. bovis* [2, 61–63]. While mainly cattle tick larvae transmit *B. bovis* and only nymphs and adult ticks have been found to transmit *B. bigemina* [58, 64], 74–90% of the larvae that infest cattle do not complete their life-cycle [65, 66], and the nymphs from surviving larvae transmit *B. bigemina*, maintaining the infection rate in cattle. Finally, although more studies are needed, some authors have reported that fetal hemoglobin contributes to the high resistance of young cattle against infection by *B. bovis* [67].

The lack of cattle challenge by *R. microplus* may interfere with the enzootic stability of the herd in terms of TF. Based on the results of a computational mathematical simulation, Smith et al. [21] reported that the high level of tick control and low inoculation rates of *Babesia* spp.*,* as induced by strategic tick control, could result in primary babesial infection in a high proportion of more susceptible adult cattle. In our study and in those conducted by Bahia et al. [39] and Martins et al. [54], although the occurrence of *B. bovis*, as measured by serology, was < 75%, there were no findings of clinical cases of babesiosis caused by *B. bovis* in adult animals. In this regard, Smith et al. [68] reported that despite the important role of ticks in establishing and maintaining herd immunity to bovine babesiosis, few studies have evaluated the effects of control strategies and tick burden on enzootic stability. According to the same researchers, this lack of data makes it difficult to determine in practice the ideal inoculation rate for *Babesia* spp. using serological data over time when tick infestation in cattle is not constant. Similarly, Bock et al. [44] reported that a detectable and persistent antibody titer is not a prerequisite for immunity, but rather is a very effective indicator of recent infection, either naturally or by vaccination. In this context, the results of the present study and of those reported by other researchers [21, 39, 44, 54] highlight the importance of performing more studies on this subject to better understand the relationship between low prevalence of *Babesia* spp. and enzootic stability in cattle herds constantly exposed to *R. microplus*, as in the present study.

Although enzootic stability for *B. bovis* was not achieved in the group treated with FLU, our results suggest that the use of this product did not affect exposure to *B. bovis* in this group, based on serology results (i.e., 40.0%). This hypothesis is supported by the serological results obtained in the control calves (46.6%) and cows (36%). In addition, all animals subjected to FLU treatment harbored ticks < 4 mm at some point during the study, and *B. bigemina,* which is transmitted mainly by nymphs of *R. microplus*, was detected in ≥ 86% of animals at 10 months of age. It is possible that the incidence of *B. bovis* in cattle on this farm is lower due to the longstanding control measures adopted against *R. microplus*. Future long-term studies should be conducted to confirm these hypotheses.

The first cases of post-weaning anaplasmosis observed in calves tend to be more severe, with higher bacteremia values and lower LBW values, which in turn may result in lower weight gain of the animals at 7 months of age [2]. Any tool that reduces these negative effects will facilitate healthier calf development and better genetic productive potential. In the present study, the group treated with FLU had lower bacteremia caused by *A. marginale* and higher PCV values (*P* ≤ 0.05) in the second and third treatments of clinical cases diagnosed after weaning. Also, FLU-treated animals presented with greater weight and weight gain at 8–9 months of age in comparison with those in the control group. These results suggest that FLU was effective in controlling cattle ticks, leading to less severe TF cases and higher animal productivity.

It is important to emphasize that our study has a number of limitations. First, the findings reported here cannot be generalized, even to farms with similar management practices, since many variables can influence enzootic stability, including tick infestation levels of calves, tick seasonality, tick infection rate with TFAs and TF treatment regimens. Another important aspect that can affect the results of tick control versus enzootic stability is the cattle breed, and our study included only Holstein cattle. In fact, the purpose of this study was not to provide generalizable information on the effect of FLU use on the enzootic stability of TFAs. Future long-term studies should be conducted on other farms in tropical, subtropical and semiarid areas. Additionally, these investigations should encompass herds that include other cattle breeds.

## Conclusions

Under the specific conditions in which this study was conducted, the use of pour-on fluralaner against *R. microplus* did not negatively affect the infection of cattle by *A. marginale* and *B. bigemina* in the study farm in comparison with the control treatment. Enzootic stability for these two TFAs occurred at 6 to 8 months of age. In contrast, the enzootic stability status was not reached for *B. bovis* in both groups, probably due to the lower *B. bovis* infection rate in the local tick population.

## Data Availability

The data supporting the findings of the study must be available within the article and/or its supplementary materials, or deposited in a publicly available database.
